# Effect of ultrasonic activation of endodontic sealers on root canal filling quality during the single-cone obturation procedure: a systematic review and meta-analysis of laboratory-based studies

**DOI:** 10.1007/s10266-025-01075-8

**Published:** 2025-04-07

**Authors:** Shuting Feng, Weiqing Zhou, Xiaojun Chu, Shuaimei Xu, Xiongqun Zeng

**Affiliations:** 1 Department of Endodontics, School of Stomatology, Stomatological Hospital, Southern Medical University, No. 366 Jiangnan Avenue South, Guangzhou, 510280 Guangdong China; 2 Department of Endodontics, Guangzhou Haizhu District Hospital of Stomatology, No. 1 and 11 Jiangnan Avenue North, Guangzhou, 510280 Guangdong China

**Keywords:** Ultrasonic, Filling quality, Calcium silicate-based sealers, resin-based sealers, single-cone technique, Root canal therapy, Obturation

## Abstract

This review aimed to investigate the potential of ultrasonic activation of sealers in improving the filling quality of single-cone obturation. A systematic search was conducted across 6 electronic databases. Data extraction and quality assessment were performed. Twenty studies were included, with 11 meeting the criteria for meta-analysis. Meta-analyses revealed lower porosity with ultrasonic activation (UA) compared to non-ultrasonic activation (NA), as demonstrated by micro-CT (MD = −1.21, 95% CI: −1.69 to 0.74, I^2^ = 28%) and stereomicroscopy studies (coronal [MD = −0.05, 95% CI: −0.82 to −0.18, I^2^ = 0%]; middle [MD = −0.44, 95% CI: −1.56 to 0.68, I^2^ = 89%]; apical [MD = −0.50, 95% CI: −0.78 to −0.21, I^2^= 0%]. Confocal laser scanning microscopy analysis showed significantly higher sealer penetration (%) with UA at the apical [SMD = 2.28, 95% CI: 1.17 to 3.40, I^2^ = 71%] and middle thirds [SMD = 2.69, 95% CI: 1.16 to 4.22, I^2^ = 87%]. However, studies examining other penetration indicators and push-out bond strength yielded inconsistent results. These findings suggest that ultrasonic activation of sealers positively influences filling quality during single-cone obturation, evidenced by decreased porosity. Further research is required to explain the variations in sealer penetration and bond strength. Ultrasonic activation of root canal sealers has emerged as a promising adjunctive technique in root canal filling. This quantitative systematic evaluation study recognizes that ultrasound-activated root canal sealers have demonstrated significant potential to achieve complete obturation in single-cone obturation.

## Introduction

Sealing both the coronal and apical portions of the tooth, as the final step in the interconnected root canal treatment procedure [1, 2], is the key to ensure the long-term effectiveness of root canal therapy [3–5]. Inadequate sealing may expose or re-expose the cleaned root canal to the risk of infection, consequently compromising the efficacy of root canal therapy [3, 5].However, achieving optimal sealing remains a challenge in current practice [6].

Single-cone obturation has become increasingly popular among dental professionals due to the rapid evolution of the physical and chemical properties of the sealer [7]. Its advantages, such as a low learning curve [8–10], time efficiency [9], low risk of heat or pressure-induced injury [11], and favorable clinical outcomes [12–16], make it an appealing option. However, doubts still remain about its effectiveness, particularly in cases where the root canals are oval or irregularly shaped [17]. This is because a larger space between the prepared canal shapes and the matching-sized gutta-percha cones can result in weaker delivery pressure of the sealer [18], increasing the likelihood of an unsealed area. Thus, there is a need to explore adjunctive methods to enhance sealer distribution along root canal walls.

To improve the versatility and clinical adaptability of single-cone obturation in root canals with varied anatomical structures, researchers have proposed several auxiliary methods, such as the use of K-files [19], Lentulo spiral [19–21], sonic activation [22], and ultrasonic activation (UA) [23]. These methods aim to achieve more effective sealing by controlling the hydrodynamic movement of root canal sealers. In recent years, UA of endodontic sealers has emerged as a promising adjunctive technique for root canal obturation [24, 25]. Existing research suggests that UA of endodontic sealers can enhance sealer distribution along root canal walls and reduce voids [18, 24, 26–28], increase bond strength between the sealer and root canal walls [29, 30], promote sealer penetration into dentinal tubules [26, 30, 31], and enhance antimicrobial efficacy and leading to a significant reduction in the viability of superficial dentin Enterococcus faecalis [32]. UA has also shown promising outcomes in the treatment of complex root canal cases. For example, Bolhari et al. [33] reported a rare case of tooth extrusion and intrusion, while Gehlot et al. [34] presented a case of non-perforating internal root resorption. Both cases were treated with UA combined with single-cone obturation and a flowable bioceramic sealer. Follow-up results indicated healing of the periapical tissues and repair of the lesions.

However, the activation of sealer through ultrasound has not always resulted in significant benefits, as some studies have found [23, 35–42]. Concerns have been raised about problems that may result from ultrasonic energy, including air incorporation, altered flow properties of bioceramic sealants after heat loss of moisture, and sealant overfilling [43–45].

To date, meta-analysis has not yet been conducted to evaluate whether UA could enhance filling obturation quality. Therefore, it is necessary to summarize and conduct an evidence-based review of current research in this field.

## Materials and methods

**Fig. 1:**
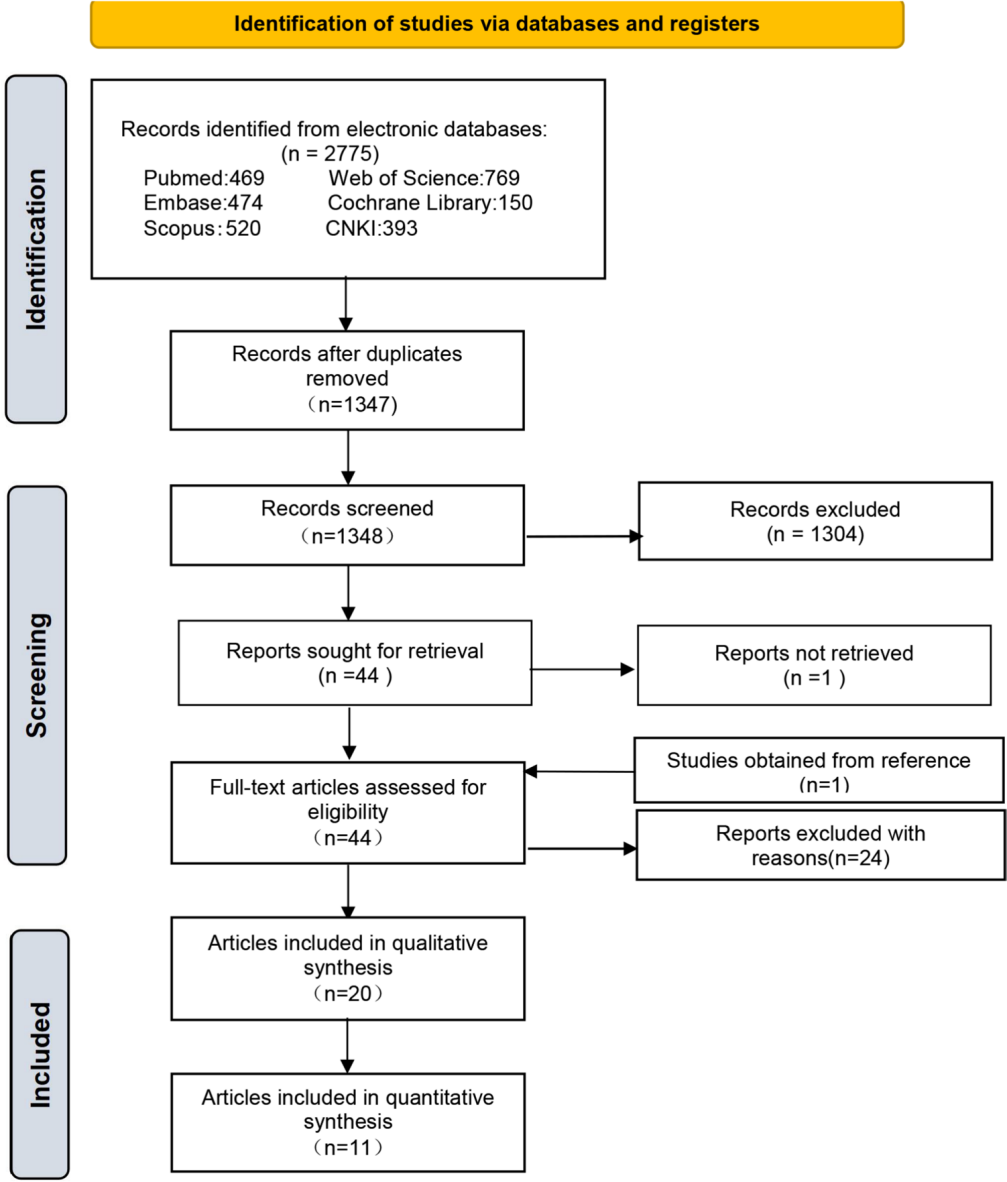
A flowchart of the literature search process

The systematic review adhered to the Preferred Reporting Items for Systematic Reviews and Meta-Analyses (PRISMA 2020) (Fig. 1) and the review was registered on the PROSPERO database with the number CRD42023406668.

### Focus question

The questions for this review were: “Does the application of ultrasound to endodontic sealers during the single-cone obturation procedure improve root canal filling quality?”

### Eligibility criteria

Studies that applied ultrasonic energy to endodontic sealers during the single-cone obturation procedure were included, and eligibility criteria were defined following the PICOS format:

- Participants (P): Extracted human teeth without immature apices or previous root canal treatment OR 3D-printed replicas of these teeth.
- Intervention (I): Single-cone obturation done using UA on endodontic sealers.
- Comparison (C): Single-cone obturation done without applying ultrasonic energy on endodontic sealers
- Outcome (O): Assessment of root canal filling quality including parameters such as porosity, sealer permeability, and bond strength.
- Study design (S): In vitro.

### Exclusion criteria

- Studies conducting with primary teeth, teeth with open canal systems, and training simulated resin blocks.
- Studies that did not obturation with single-cone technique or UA.
- Studies that evaluated the application of ultrasonic energy on root canal irrigation, apicectomy, retrograde filling, the compaction of gutta-percha, and the removal of root canal filling materials
- Those that did not evaluate the quality of root canal filling, those that focused solely on microleakage assessment [46, 47], and those that failed to specify the measurement method.
- Narrative reviews, case reports, opinion pieces, conference abstracts, and letters to the editor.

### Search strategy

A search was carried out in the following six electronic databases: PubMed, Embase, Web of Science, Cochrane Library, Scopus, and China National Knowledge Infrastructure (CNKI, https://www.cnki.net/). The final search was conducted in December 2024. The details of the search strategies defined for each database are given in Table 1.

**Table 1:** Search strategy

Database	Search details (December, 2024)	Results
PubMed	(((((Ultraso*)) AND (Endodontic OR (root canal))) AND (sealer OR sealant OR (Root Canal Filling Materials))) AND (((obturation OR filling OR sealing)) OR (Root Canal Obturation))) NOT ((irrigant*[Title]) OR irrigation[Title] OR ("Root Canal Irrigants/administration and dosage"[MeSH Terms]))	469
Web of science	(TS=(((((Endodontic OR (root canal))) AND (Ultraso*)) AND (sealer OR sealer OR (Root Canal Filling Materials))) AND (obturation OR filling OR sealing OR (Root Canal Obturation) OR (single-cone technique)))) NOT TI=((irrigant*) OR irrigation)	769
Scopus	TITLE-ABS-KEY( ( ( endodontic* OR ( root AND canal ) ) AND ( ultraso* ) AND ( sealer OR sealant OR ( root AND canal AND filling AND materials ) ) AND ( ( obturation OR filling OR sealing ) OR ( root AND canal AND obturation ) OR ( single-cone AND technique ) ) ) ) AND NOT ( TITLE ( ( irrigant* ) OR irrigation ) )	520
Embase	(endodontic* OR 'root canal*':ti,ab,kw) AND ultraso* AND (sealer*:ti,ab,kw OR sealer*:ti,ab,kw OR 'root canal filling material'/exp OR 'root canal filling material*' OR (('root'/exp OR root) AND canal AND filling AND material*)) AND (obturation*:ti,ab,kw OR filling*:ti,ab,kw OR sealing*:ti,ab,kw OR 'root canal'/exp OR 'root canal' OR (('root'/exp OR root) AND canal AND obturation*) OR 'single-cone technique' OR ('single cone' AND ('technique'/exp OR technique))) NOT (irrigation*:ti OR irrigant*:ti)	474
Cochrane library	ALL Text: ((endodontic*) OR (root canal)) AND (ultraso*) AND ((sealer*) OR (sealer*) OR (material*)) AND ((obturat*) OR (fill*) OR (seal*) OR (single-cone technique))	150
CNKI^1^	all text :("Gen Guan") and all text :("Chao Sheng") and all text :(Feng Bi Ji) and all text :("Chong Tian")	393

^1^China National Knowledge Infrastructure (CNKI, https://www.cnki.net/)

### Study selection

The results obtained from these queries were exported to Zotero. After the removal of duplicates, two reviewers (ST, XJ) independently screened the titles and abstracts of all articles according to the above criteria. Full texts of potentially eligible studies were obtained and assessed. Any disagreements were resolved by consensus or in consultation with a third reviewer (WQ).

### Data extraction

Data from each study were extracted and organized in Excel, with key information systematically tabulated and integrated into the main text or presented as supplementary material. The Excel items included: author (year), sample type and size, specifics of root canal preparation (including instrumentation systems, apical sizes, and irrigation protocols), details of root canal filling (sealer type and delivery method, gutta-percha cones used, and storage conditions), protocols for UA (tip type and size, device settings, tip placement, and activation duration), the assessment method, as well as the main parameter and results. Two reviewers (ST, WQ) performed the data extraction independently and disagreements were resolved through discussions or with the assistance of a third reviewer (XJ). Authors of the included studies were also approached via e-mail for missing necessary data and uncertainty.

### Quality assessment and risk-of-bias analysis search strategy

The risk-of-bias assessment of the included studies was conducted based on the method used in previous systematic reviews [6, 48] with adjustments. The assessment items were as follows: (i) sample-size calculation, (ii) samples with similar dimensions, (iii) teeth randomization, (iv) standardization of instrumentation procedures, (v) standardization of filling procedures, (vi) endodontic treatment performed by a single operator, (vii) blinding of sampling and assessment, and (viii) statistical analysis and other bias.

Each item of each included studies was judged as “low” (green dot), “high” (red dot) or “unclear” (yellow dot) risk of bias. The assessment was conducted independently by two trained reviewers (ST and WQ). Disagreements were resolved through discussion or with the involvement of a third reviewer (XJ). Each included study was given an overall judgment according to the risk of bias of each domain:

- Low risk of bias: studies that had 7–8 items with low risk of bias;
- Moderate risk of bias: studies that had 4–6 items with medium risk of bias;
- High risk of bias: studies that had less than 4 items with high risk of bias.

### Data synthesis and meta-analysis

Meta-analyses were performed separately according to the outcomes of different indicators, after aggregating the mean ± standard deviation (SD) and sample size of each interest group in studies with methodological homogeneity. For studies that reported data using order statistics (such as median, minimum and maximum, and first and third quartiles), a qualitative analysis was performed instead of conducting a meta-analysis. We chose to calculate the mean difference (MD) and corresponding 95% confidence interval (95% CI) as the meta-effect estimate if the same measurement tools and units were used across studies; otherwise, we calculated the standardized mean difference (SMD) and corresponding 95% confidence interval (95% CI) for each eligible study. Statistical heterogeneity was evaluated using the Chi-square test and I^2^ test, and subgroup analyses were conducted to explore the reasons behind the observed heterogeneity among studies. These comparisons were performed utilizing a random-effects model.

All analyses were performed using Review Manager Software (RevMan 5.3.4).

## Results

### Study selection

The search process is depicted in Fig. 1. A comprehensive database search yielded a total of 2774 records. Following the removal of duplicates, we screened 1347 articles based on their titles and abstracts using the eligibility and exclusion criteria through the PICO strategy, resulting 44 records selected for full-text evaluation. One report could not be retrieved [49], and one additional study was obtained through a comprehensive manual search of the references list [50]. After assessing all 44 full texts, we excluded 24 articles [18, 24, 26, 27, 29, 31, 42, 45, 51–66] for various reasons specified in Table 2. Ultimately, a total of 20 in vitro articles [23, 32, 35–41, 43, 44, 50, 67–72] published between the years 2015 and 2024 were included in the review.

**Table 2:** Excluded studies and reasons for exclusion

Reasons for exclusion	References
Inappropriate study group	Aguiar et al. 2019 [26]; Cho 2021 [18]; Cho, Kwak, and Shin 2021 [27]; Lee et al. 2022 [61]; Pérez-Alfayate et al. 2021 [59]; Barreto et al. 2023 [63]; Küçükkaya Eren S et al. 2019 [56]; Tanomaru-Filho et al. 2024 [42]
Inappropriate control group	Hwang et al. 2015 [31]; Cakici et al. 2016 [29]; Yazdi 2018[54]; Song and Yang 2022 [45]
Not using single cone obturation	Aguirre, Deeb, and Aguirre 1997 [51]; Nikhil and Singh 2013 [52]; Guimarães et al. 2014 [24]; Ramlan et al. 2020 [57]; Bhor et al. 2023 [62]
Vague details of obturation or measurement	Chandrasekhar et al. 2016 [53]; Prasad et al. 2018 [55]; Gonçalves et al. 2021 [58]; Yamini et al. 2021 [60],Mohammed et al. 2021 [64]
Assess apical microleakage	Alafandi et al. 2021 [65] Long et al. 2018 [66]

### Characteristics of the included studies

The characteristics of all the 20 included studies are listed in Table 3.

**Table 3:** The characteristics of all the 20 included studies

Indicators	Author, Year	Testing Samples(type and size)	Sealer	Ultrasonic activation method	Method	Main parameter	Main results
Porosity	Drukteinis et al. 2021 [43]	moderately curved (10°–20°), two separate mesial canals of mandibular first molars with artificial simulated apical perforations (*n* = 20)	Bioceramic-based	Direct 10s	Micro-CT(9.9 μm pixels )	void volume % (Mean ± SD) (apical 5mm)	BioRoot RCS: no significant difference between UA* and NA* at apical 5mm regarding open and closed Pores
	Kalantar Motamedi et al. 2021 [44]	straight, Vertucci type I, mostly round canals of mandibular first premolar (*n* = 9)	Epoxy resin-based	Indirect 3s	Micro-CT(19μm pixels )	void volume % (Mean ± SD) (apical ,middle, coronal ,overall)	AdSeal: significantly lower void volumes in UA than in NA at all three levels
	Artingsih et al. 2021 [72]	Circular canals of single-rooted first mandibular premolar (*n* = 8)	Bioceramic-based	Indirect 2~3s	Micro-CT(13.47 μm pixels )	void volume % (Mean ± SD) (overall)	1.iRoot SP: significantly lower void volumes in UA than in NA 2.Endoseal MTA :no significant difference between UA and NA.
	Sfeir et al. 2023 [40]	straight( < 5°), Vertucci type I canals of single-rooted first mandibular premolars (*n* = 9)	Bioceramic-based	Indirect 3s	Micro-CT(40μm pixels )	void volume % (Mean ± SD) (apical ,middle, coronal ,overall)	TotalFill BC: no significant difference between UA and NA at all three levels.
	Kim et al. 2021 [69]	oval-shaped canals of single-rooted premolars (*n* = 12)	Bioceramic-based	Indirect 2~3s	Micro-CT(9.94μm pixels)	void volume % (no data) (apical ,middle, coronal ,overall)	EndoSequence BC: significantly lower void volumes in UA than in NA at all three levels
	Kim et al. 2018 [23]	ribbon-shaped canals of single-rooted maxillary premolars containing 2 canals (*n* = 10)	Bioceramic-based	Indirect 2~3s	Micro-CT(30 μm pixels )	void volume % (Mean ± SD) (overall)	Endoseal MTA: no significant difference between UA and NA.
	Loiacono et al. 2024 [74]	straight, single-rooted mandibular premolars with a single root canal (*n* = 10)	Bioceramic-based	Indirect 3s×3^a^	Micro-CT(12 μm pixels)	void volume % (Mean ± SD) (apical ,middle, coronal ,overall)	Bio-C sealer: no significant difference between UA and NA at all three levels , with the coronal third having a higher percentage of voids than the middle and apical third
	Ko et al. 2020 [68]	ribbon-shaped canal of single-rooted teeth (*n* = 10)	Bioceramic-based	Indirect 3s	Micro-CT(30 μm pixels)	Root filling volume (%)(Mean ± SD) (overall)	Endoseal TCS: no significant difference between UA and NA.
					stereomicroscope+ 4-grade scoring system^b^	void scores + void number(Mean ± SD) (overall)	Endoseal TCS: significantly lower number and score of voids in UA than NA.
	Damade et al.2020 [36]	single-rooted mandibular premolars (*n* = 10)"	Bioceramic-based	not provided	stereomicroscope+ 4-grade scoring system	Void scores(Mean ± SD) (apical ,middle ,coronal)	1.Endoseal MTA : no significant between UA and NA at all three levels. 2.GuttaFlow Bioseal :no significant between UA and NA at all three levels. 3.Endoseal MTA VS GuttaFlow Bioseal :no statistically significant difference was found at all three levels , with or without UA.
	Moazami et al. 2020 [39]	straight canal of anterior single-root teeth (*n* = 12)	Bioceramic-based	Indirect 2~3s	dental microscope + 4-grade scoring system	void scores (not provided)and void area(μm2) (Mean ± SD) (apical ,middle, coronal ,overall)	Endoseal MTA :no significant difference in void area.
	Gharechahi et al. 2024 [73]	3D-printed replicas of human mandibular molars with C1 anatomy (*n* = 10)	Bioceramic-based	Indirect 2~3s	stereomicroscope	sealer and void areas (%) (Mean ± SD) (apical, middle, coronal)	1.EndoSeal TCS: significantly less voids areas in UA than NA at middle and coronal level. No significant difference in apical level. 2.Endoseal MTA : significantly less voids areas in UA than NA at middle and coronal level. No significant difference in apical level.
	Alcalde et al. 2017 [32]	Vertucci type IV canals with Vertucci type V isthmus of mandibular first molars (*n* = 15)	Epoxy resin-based	Direct 20s	stereomicroscope	unfilled areas (%) of the canal and isthmus( Median, Min, Max)	AH Plus: significantly less unfilled areas in the canal and isthmus areas in UA than NA at all three levels
					CLSM*	gaps percentages (%) of the canal and isthmus( Median, Min, Max)	isthmus: significantly less gaps at 2 and 6 mm in UA than NA. Canal: significantly less gaps at 6 mm in UA than NA
	Carneiro et al. 2023 [70]	curvatures ≤20° ,distobuccal root canals of maxillary molars (*n* = 12)	Bioceramic + Epoxy resin-based	Direct 40s	CLSM	interfacial gaps %(Median, Min, Max)	1.EndoSequence BC : significantly less gaps in UA than NA at 6 mm level. 2.Sealer Plus BC: significantly less gaps in UA than NA at all three levels. 3.Bio-C Sealer: significantly less gaps in UA than NA at 2,4 mm level. 4.AH Plus: significantly less gaps in UA than NA at 4 mm level.
Sealer penetration	Alcalde et al. 2017 [32]	Vertucci type IV canals with Vertucci type V isthmus of mandibular first molars (*n* = 15)	Epoxy resin-based	Direct 20s	CLSM Rhodamine-B dye (0.1 wt%) Area of testing: 2,4, 6mm from root apex	percentage of perimeter penetrated of the canal and isthmus( Median, Min, Max)	AH Plus :significantly improve sealer penetration in both canals and isthmuses with UA than NA at 2,4,6 mm level
	Carneiro et al. 2023 [70]	curvatures ≤20° ,distobuccal root canals of maxillary molars (*n* = 12)	Bioceramic + Epoxy resin-based	Direct 40s	CLSM Fluo-3 (0.1 wt%) Area of testing: 2,4, 6mm from root apex	average depth of penetration(mm)(Median, Min, Max)	1.EndoSequence BC 2.Sealer Plus BC 3.Bio-C Sealer 4.AH Plus no significant difference at 2,4,6 mm level between all groups.
	Dash et al. 2017 [37]	single-rooted maxillary teeth with one canal (*n* = 10)	Epoxy resin-based	Direct 5s	CLSM Rhodamine-B dye (0.1 wt%) Area of testing: 3, 6mm from root apex	percentage of sealer penetration (%) (Mean±SD)	Adseal: significantly decrease sealer penetration in Group UA than Group bidirectional spiral at level 6 ,Group Lentulo spiral at level 3.
						average depth of penetration(μm)(Mean±SD)	Adseal: no significant differences between groups at 3 and 6mm levels.
	Nikhil et al. 2015 [67]	curvature<10°, single canal of maxillary central incisors (*n* = 20)	Bioceramic + Epoxy resin-based	Direct 20s	CLSM Rhodamine-B dye (0.1 wt%) Area of testing: 3, 6mm from root apex	maximum depth of penetration(μm)(Mean±SD)	1.AH plus: significantly improve sealer percentage and depth of penetration at 3, 6mm from root apex 2.MTA Fillapex: significantly improve sealer percentage and depth of penetration at 3, 6mm from root apex 3.Irrespective of method used for agitation, the percentage and depth of sealer penetration for MTA Fillapex was significantly greater than AH plus.
						percentage of sealer penetration (%) (Mean±SD)	
	Keles et al. 2023 [38]	round-shaped root canals of single-rooted teeth (*n* = 15)	Bioceramic-based	Direct 20s	CLSM Rhodamine-B dye (0.1 wt%) Area of testing: 5 mm from root apex	percentage of sealer penetration (%) (Mean±SD)	Sure-Seal RootTM sealer:no significant difference at 5 mm level between groups.
	Coronas et al. 2020 [35]	curvature<5° ,distobuccal roots of superior molars with three distinct roots (*n* = 10)	Bioceramic-based	Direct 30s	CLSM Fluo-3 (0.1 wt%) Area of testing: 2, 7 mm from root apex	percentage of the sealer penetration area(% ) ( Median, 25 and 75 percentile)	Sealer Plus BC: no significant difference at 2, 7 mm between groups.
	Maharani, Ricardo, and Artiningsih 2021 [71]	straight,single canal of premolar teeth (*n* = 8)	Bioceramic-based	Indirect 3s	CLSM Rhodamine-B dye (0.1 wt%) Area of testing: 5 mm levels from the apical foramen"	maximum depth of penetration(μm)(Mean ± SD)	IRoot SP: no significance diference at 5 mm from root apex. Endoseal MTA: significantly improve sealer depth of penetration at 5 mm from root apex.
Push-out bond strength	Carneiro et al. 2023 [70]	curvatures ≤20° ,distobuccal root canals of maxillary molars (*n* = 12)	Bioceramic + Epoxy resin-based	Direct 40s	•Storage condition : 100% humidity at 37 °C for 7 days • The thickness of each slice was measured with a digital caliper • the three-thirds root(2、4、6mm) • Plunger size: chosen according to the diameter of the root canal.	push-out bond strength(MPa) (Median, Min, Max) (apical, middle, coronal)	1.EndoSequence BC sealer: no significant difference between UA and NA at 2,4,6mm level. 2.Sealer Plus BC : significantly higher bond strength in UA than NA at 2 mm level. 3.Bio-C sealer: significantly higher bond strength in UA than NA at 6 mm level. 4.AH Plus: no significant difference between UA and NA at 2,4,6 mm level.
	Chadgal et al. 2018 [50]	curvatures <20° ,single-rooted mandibular premolars (*n* = 40)	Bioceramic-based	Direct 60s	•Storage condition : 95% humidity at 37 °C for 14 days • 1.0mm-thick slices • 2 thirds(the coronal, apical parts) • Plunger size : diameters with 0.35, 0.5, 0.65, 0.8, 1.0mm ( provided 75 to 80% coverage of intracanal material without touching the circumferential dentin) Cross head speed of 1 mm/min	push-out bond strength(MPa) (Median, values) (apical, coronal)	EndoSequence BC sealer: significantly higher bond strength in UA than NA at apical and coronal levels.
	Tanita et al. 2021 [41]	single-rooted mandibular premolars with one root canal (*n* = 8)	Bioceramic-based	Indirect 3s	•Storage condition : 100% humidity at 37 °C for 1 days • 2.0mm-thick slices • middle third (at 5-7 mm from the apex ) • Plunger size : 0.6 mm. Cross head speed :not provided	push-out bond strength(Mpa)(mean ± SD) (middle)	1.Endoseal MTA: no significant difference between UA and NA at middle levels. 2.IRoot SP : no significant difference between UA and NA at middle levels. 3. significantly higher bond strength in Endoseal MTA than IRoot SP .

*Direct,* the direct ultrasonic activation methods place the ultrasonic tips directly into the canal, primarily 2mm short of the working length

*Indirect,* the indirect ultrasonic activation methods place the ultrasonic tips against the cotton pliers holding the gutta-percha cone during insertion of the gutta-percha cone

*UA* ultrasonic activation ; *NA* non-ultrasonic activation ; *n* sample size; *SD* standard deviation; *Min* minimum; *Max* maximum ; *wt%* weight percent ; *CLSM* confocal laser scanning microscope

^a^*3s×3,* Ultrasonic vibration was used for 3 periods, each period for 3 seconds

^b^*4-grade scoring system,* 4-grade scoring system provides a quantitative assessment of root canal filling quality by evaluating the number and size of voids, with scores from 1 (minimal small bubbles) to 4 (numerous and/or large void)[23]

Thirteen studies [23, 32, 36, 39, 40, 43, 44, 68–70, 72–74] evaluated the effect of ultrasound on porosity within filling canal. Seven studies [32, 35, 37, 38, 67, 70, 71] assessed the dentinal tubule penetration of sealers. Three studies [41, 50, 70] on the push-out bond strength (PBS).

The literature sample included a variety of canal structures in permanent human teeth, including round, flat, ribbon canals, and canals with isthmus structures. Additionally, one study employed artificially simulated apical perforations [43], while another sample was derived from a 3D-printed replica of a human mandibular molar with a C1 anatomical structure [73].

In the single-cone obturation process, a single gutta-percha cone matching the taper and size was used for each prepared root canal, without the addition of any accessory points.

The UA methods can be categorized into two types: direct and indirect, as shown in Appendix A. The operational details of the indirect method are relatively consistent, with an activation time of 2~3 s. The commonly used ultrasonic tip is StartX #3 (Dentsply-Maillefer), paired with the P-5 Newtron XS device (Satelec). The power setting for this equipment is typically set to "8" in the yellow code. On the other hand, the direct method generally uses an activation time of about 20 s. Although it may vary between 5 s and up to 60 s in certain cases. Ultrasound devices are typically set in the mid-to-low power range of their respective devices. The tip placement position is primarily 2 mm short of the working length (WL).

The sealers investigated included two resin-based sealers, predominantly AH Plus, and nine calcium silicate-based sealers, with the latter primarily consisting of Endoseal MTA, iRoot SP/TotalFill BC/EndoSequence BC, as shown in Appendix B.

### Outcomes of different indicators

### Voids and gaps

Thirteen included articles [23, 32, 36, 39, 40, 43, 44, 68–70, 72–74] compared voids and gaps within filled root canal between UA and NA groups.

Eight studies [23, 40, 43, 44, 68, 69, 72, 74], comprising nine groups of sealers, were evaluated using micro-CT. The results revealed significant lower void volumes in UA than in NA in three groups, while no significant differences in the remaining six groups. Among them, 6 studies including 7 groups of sealers were quantitatively synthesized through meta-analysis.

Five articles [23, 36, 39, 68, 74] employed microscopic to assess the voids of 8 groups of sealers, resulting in 6 groups displaying significant differences, while 2 groups showed no significant differences between UA and NA. Two articles [39, 73] were excluded from the meta-analysis due to limited microscope resolution and differing outcome measures, respectively. Quantitative analysis was performed on the remaining 3 articles [23, 36, 68]

Two studies utilized confocal laser scanning microscopy (CLSM) to examine interfacial gaps between the root canal walls and the sealer. Alcalde et al. 2017 [32] reported that UA of AH Plus sealer significantly reduced unfilled areas, especially in the isthmus region (p<0.05), whereas Carneiro et al. (2023) [70] showed that UA markedly enhanced the marginal adaptation of all investigated sealers (p<0.05).

**Fig. 2:**
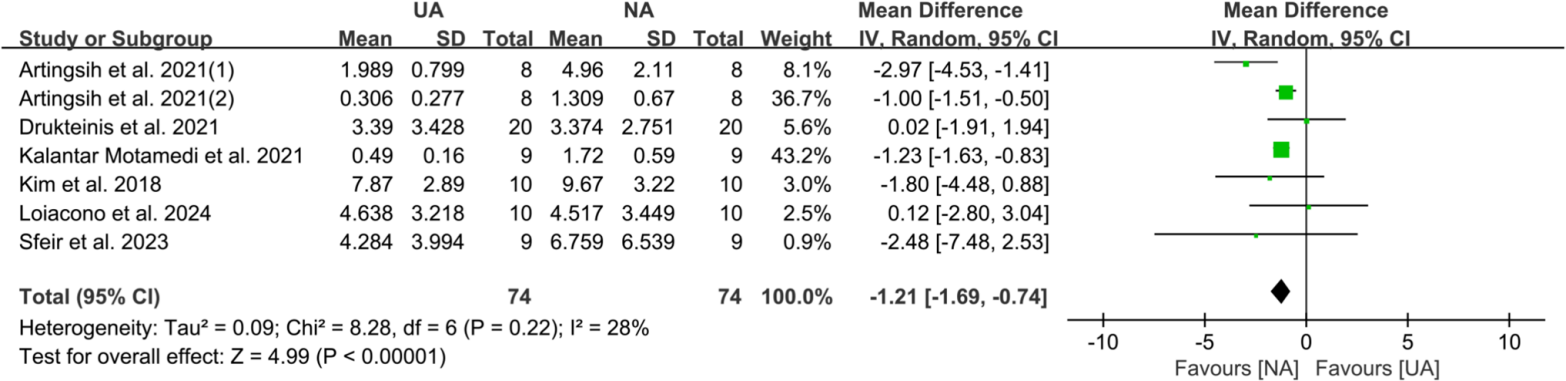
Forest plot of void volume percentages (%) on the entire root canal space comparing UA with NA by micro-CT evaluation(3D) . Abbreviations. *UA,* ultrasonic activation ; *NA,* non-ultrasonic activation

**Fig. 3:**
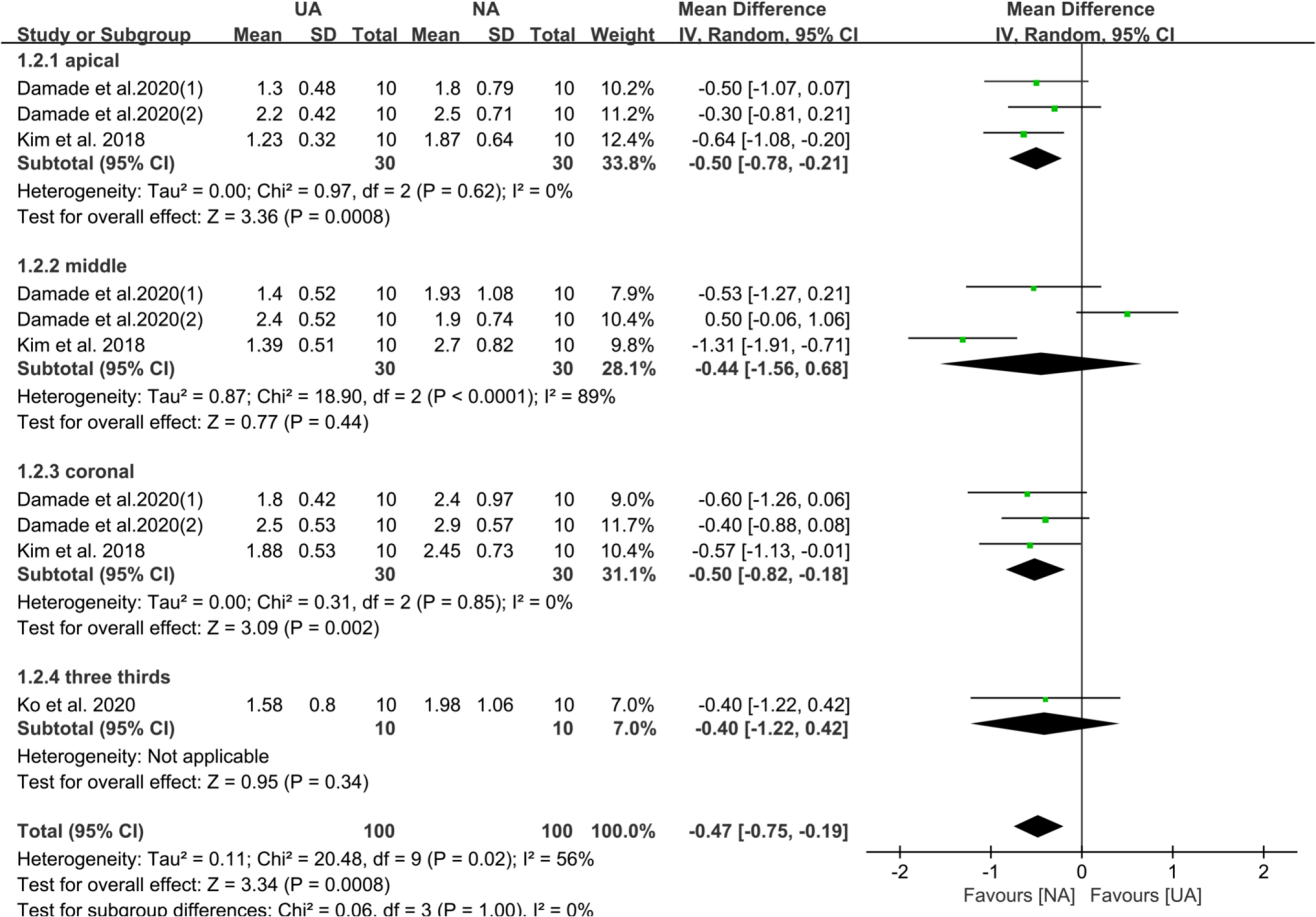
Forest plot of void score of each sectioned specimen comparing UA and NA by stereomicroscope evaluation in apical, middle, coronal and three thirds. Abbreviations. *UA,* ultrasonic activation ; *NA,* non-ultrasonic activation

The meta-analyses conducted on the entire root canal space using micro-CT (3D) revealed significantly lower percentages of void volumes in the UA group compared to the non-ultrasonic activation (NA) group (MD = −1.21, 95% CI −1.69 to 0.74, I^2^ = 28%) (Fig. 2). Similarly, the meta-analyses performed on each slice region evaluation by stereomicroscopy (2D) also demonstrated significantly lower void scores in each sectioned area of the UA group compared to the NA group. (coronal [MD = −0.05, 95% CI −0.821 to −0.18, I^2^ = 0%]; middle [MD = −0.44, 95% CI −1.56 to 0.68, I^2^ = 89%]; apical [MD = −0.50, 95% CI −0.78 to -0.21, I^2^ = 0%] (Fig. 3)

### Dentinal tubule penetration

Seven studies compared sealer dentin permeability between UA and NA groups using confocal laser scanning microscope (CLSM) [32, 35, 37, 38, 67, 70, 71].

Depth of sealer penetration (μm) was evaluated in 9 groups of sealers in 4 studies [23, 36, 39, 68], with 2 studies finding no difference between groups in the average depth(μm) among 5 groups of sealers , and the other 2 studies indicating statistically significant improvement with UA in maximum depth (μm) of 3 groups among 4 groups. Meta-analyses were not performed as several studies reported outcome data as order statistics (i.e., median, interquartile range, and/or maximum–minimum values).

Area of sealer penetration (%) was evaluated in 1 study showing no significant differences between UA and NA [35].Percentage of canal perimeter penetrated (%) was assessed in 1 study and showed a significant improvement with UA [32].

**Fig. 4:**
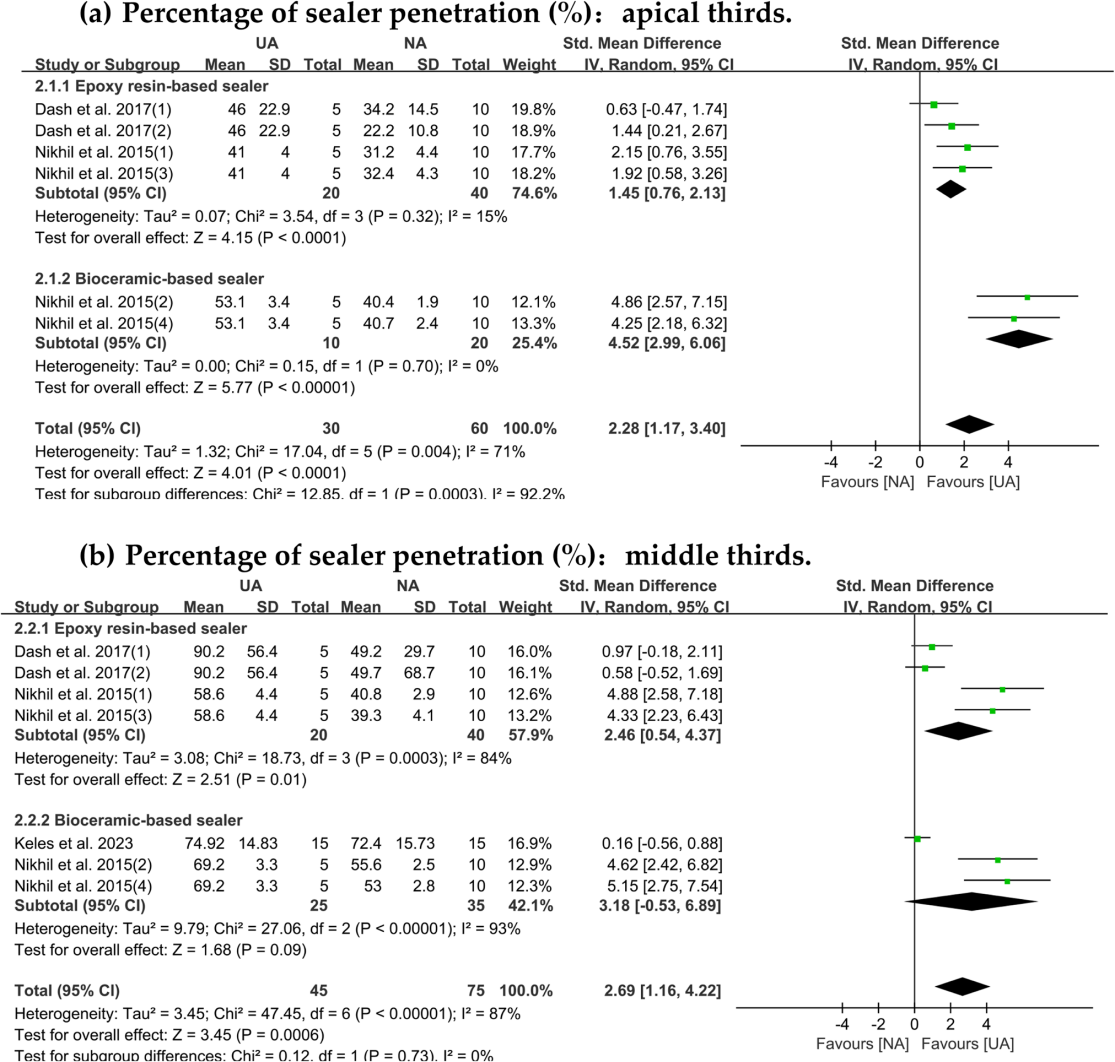
Forest plot of percentage of sealer penetration (%) comparing UA with NA in apical (**a**) and middle (**b**) thirds using CLSM Evaluation. *UA, ultrasonic activation; NA, non-ultrasonic activation*; *CLSM* confocal laser scanning microscope)

Percentage of sealer penetration (%) was assessed in 3 studies [37, 38, 67] involving 4 groups of sealers, and then quantitatively synthesized through meta-analysis. This analysis revealed that UA significantly improves sealer penetration rates at the apical and middle third of the root canal (apical [SMD = 2.28, 95% CI: 1.17 to 3.40, I^2^ = 71%]; middle [SMD = 2.69, 95% CI: 1.16 to 4.22, I^2^ = 87%]. Subgroup analysis further highlights improvements in most root canal segments, regardless of the sealer type used (Fig. 4a, b).

#### Push-out bond strength

Three studies evaluated the push-out bond strength (PBS) in MPa of seven groups of sealers [41, 50, 70]. The results indicated no significant difference in four of the groups, while the remaining three groups showed significantly higher bond strength in certain root canal cross sections with UA.

### Quality assessment

**Fig. 5:**
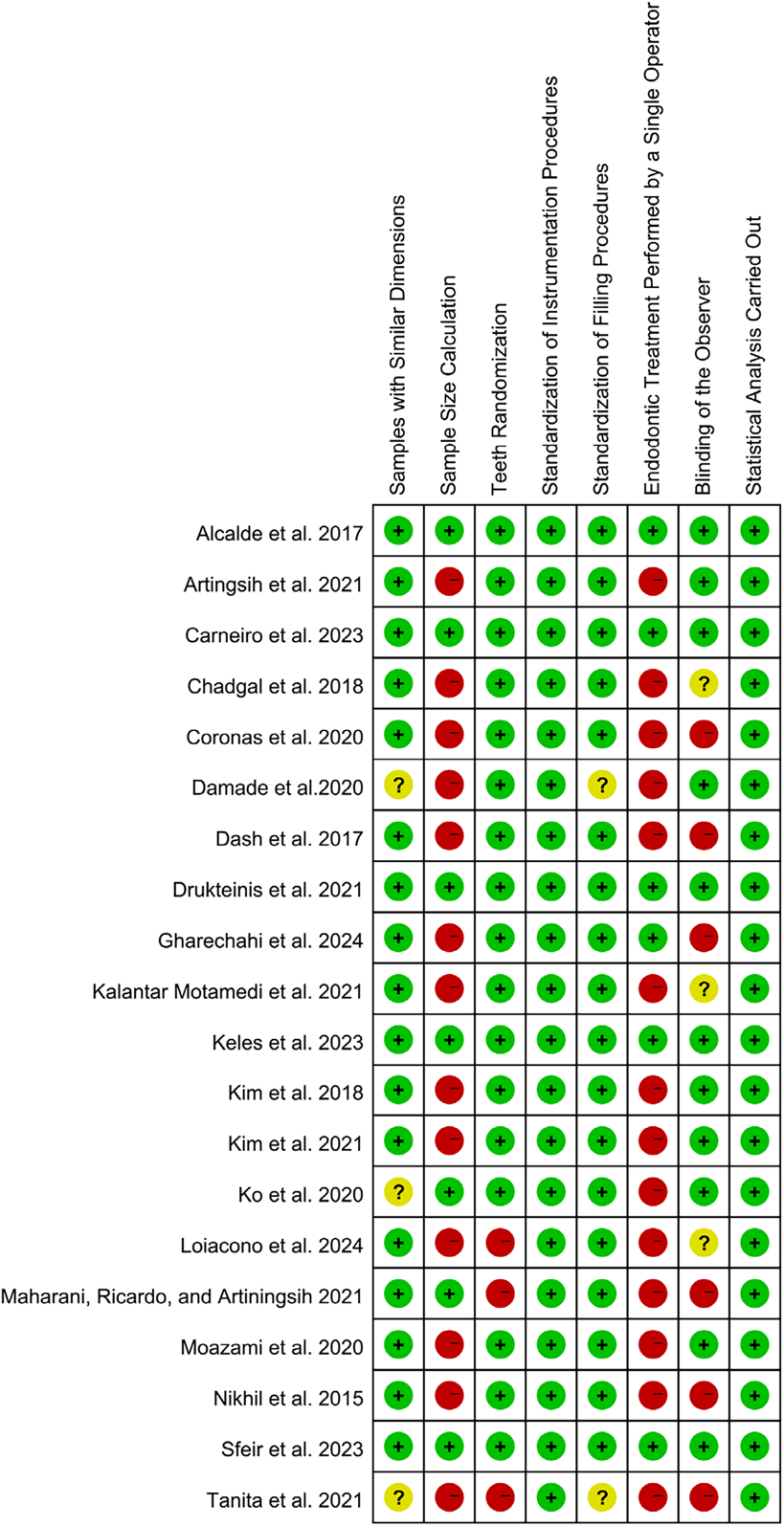
Risk of bias of each included study

**Fig. 6:**
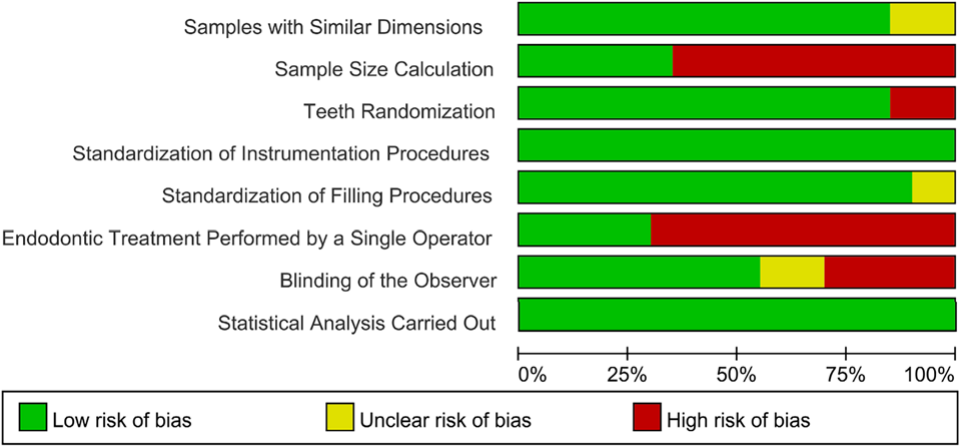
Summary of risk of bias

The quality assessment of the 20 articles that met the inclusion criteria is shown in Figs. 5 and 6. The overall assessment of potential bias in the studies included was classified into different categories: low risk (*n* = 6) [32, 38, 40, 43, 68, 70], medium risk (*n* = 13) [23, 35–37, 39, 44, 50, 67, 69, 71–74], and high risk (*n* = 1) [41]. The main limitations of the included studies were the absence of a sample-size calculation(*n* = 13) , failure to mention a single operator conducting all procedures (*n* = 14) , and lack of blinding in sampling and outcome assessments (*n* = 6) .

## Discussion

To the authors' knowledge, this is the first systematic review and meta-analysis to compare the effect of UA of sealers versus NA on filling quality. Under the current limitations, the overall results suggest that the application of ultrasonics has a positive effect on improving root canal filling quality during the matched single-cone obturation procedure.

Notably, this review focuses on comparing UA and NA using the matched single-cone obturation technique. The exclusion of studies employing thermoplasticized gutta-percha techniques and cold lateral condensation techniques is intended to increase the reliability of results by reducing uncontrollable variables related to pressure dynamics, such as operator variances in clinical experience and thermoplastic techniques [75], which could interfere with the assessment of ultrasound effects.

In addition, heat produced from thermoplasticized gutta-percha techniques may affect the performance of the bioceramic sealer [76, 77] . Therefore, we will focus on the single-cone filling technique, enabling obturation with a standardized quantity of gutta-percha. This approach simplifies the standardized procedure and steps, thereby reducing variability. It also reduces the influence of clinical experience [8–10], thus minimizing the potential for bias and confounding variables.

### Porosity

The presence of porosity within the obturated root canal poses a potential risk, as they might serve as pathways and shelters for microorganisms, as well as channels for the infiltration of fluids [78, 79]. Especially, when employing single-cone obturation in larger or irregular root canals, the required sealer volume around the master cone is often greater [79], potentially leading to uneven sealer distribution, residual gas entrapment, and gap formation [17, 44, 80].

In general, the application of UA to sealers appears to effectively reduce voids and gaps within the sealer and between the sealer and the root canal walls. This reduction may be attributed to ultrasonic energy inducing rapid compression vibration of sealer particles [23, 81], leading to the overflow of gas accumulated in the root canal or carried in the sealer [23, 40]. Additionally, some studies suggests that it simultaneously reduces the cement particle size [82], decreases interparticle friction [68], and promotes a more uniform polymerization reaction [28], thereby promoting a homogeneous and densely packed curing state [43, 82]. Moreover, the supplementary pressure facilitates coverage of challenging-to-access areas by the filling material [32].

The moderate heterogeneity observed in the micro-CT meta-analysis could be attributed to variations in sealer type and ultrasound application. Regarding the type of sealer, most of the 4 studies used calcium silicate-based sealer, while 1 study used AdSeal, an epoxy resin-based sealer [44]. Furthermore, differences were also observed among various types of calcium silicate-based sealer. Artingsih et al.'s study [72] found contrasting results between iRoot SP and Endoseal MTA regarding void volume under UA. iRoot SP showed a notable decrease in void volume, whereas Endoseal MTA showed no significant change between UA and NA. Additionally, the void volume of the Endoseal MTA group was consistently smaller than that of the iRoot SP group irrespective of UA. This is likely attributable to its alumina-silicate composition, which reacts with water to induce a pozzolanic reaction, resulting in a material that is uniformly dense with minimal detectable voids [71]. Research has found that the compositions of sealers, including polymerization agents, viscous carrier type, and particle size, have an impact on ultrasound effectiveness [42, 60, 83–85] . However, there is limited literature regarding the influence of UA on the physicochemical properties of root canal sealers [60, 81, 83, 84], necessitating further research. Additionally, a comprehensive investigation into the activation effects of different types of sealers is also needed. In terms of ultrasonic application methods, the primary difference between direct and indirect ultrasonic techniques lies in how energy is transmitted. Direct activation delivers ultrasonic energy directly to the sealer, improving its filling capability but also brings potential risks. Brief activation periods can produce impact-like effects [43, 86], whereas prolonged activation durations may elevate temperature and result in moisture loss within filling materials [43, 81, 84], among other concerns. Conversely, indirect UA promotes a milder and homogeneous energy transmission, reducing the risk of air entrapment and sealer overfilling, and minimizing the negative effects of temperature variations [23, 64]. However, there is a lack of comparative studies on the effectiveness between direct and indirect activation methods and no specific guidelines for the application of ultrasonic energy; further investigation is needed to determine their actual efficacy. In the meta-analysis using a stereomicroscope (Fig. 3), high heterogeneity (I^2^ = 89) was observed specifically in the middle segment of the root, likely attributable to the anatomical variations among maxillary [23] and mandibular premolars [36].

The studies reviewed in this section exhibit a moderate-to-low risk of bias, primarily due to the absence of sample-size calculations and insufficient information on a single operator. Nevertheless, the quantitative analysis overcame the limitation of sample size and comprehensively considered two different research methods: micro-CT and stereomicroscopy. Stereomicroscopy allows clear visualization of the microscopic structure and tiny voids [87]. However, inevitable material loss during the sample cutting process [69] and limitations in cross-sectional information may affect result accuracy [88] . Micro-CT presents distinct benefits in voids and gaps observation due to its exceptional spatial resolution and non-invasive scanning properties. It utilizes an automated analysis for accurate localization and quantification of target voids, thus diminishing potential observer bias [6, 43, 89]. However, practical application may be challenging [90]. The high radiopacity of sealers [91], combined with the limitations of voxel resolution and image processing techniques [92], results in the generation of artifacts and noise, thereby reducing sensitivity to void detection. The findings of Kim et al. [23] and Ko et al. [68] suggest that CT imaging may be less sensitive than stereomicroscopy, partly due to the micro-CT scanners used by both teams having a resolution of only 30 μm, which is lower than the recommended 11.2 μm [93]. Further analysis revealed that out of the 8 studies included in the overall literature, 6 failed to meet this requirement, which may have led to inadequate detection of voids. This may also explain why the majority of the results in the micro-CT section of the studies show no significant differences. Kim et al. [23] suggests that it is necessary to use microscopic observation of sliced specimens as an auxiliary means of evaluation. Considering the above, integrating both micro-CT and stereomicroscopy enhances the comprehensiveness and accuracy of the analysis. Future research could explore the use of higher-resolution micro-CT techniques, such as phase contrast enhanced (PCE) micro-CT [6]. Additionally, incorporating the element of time into the investigation.

### Dentinal tubule penetration

Increased sealer penetration into dentinal tubules is deemed beneficial for improving the sealing of the root canal system [94]. UA significantly improves the penetration of dentinal tubule sealers, as demonstrated by the results of the meta-analysis (Fig. 4).

The observed heterogeneity and inconsistencies may arise from the following factors. First, different types of sealers may contribute to these variations. For instance, the penetration rate and depth of MTA Fillapex are significantly superior to those of AH Plus, regardless of the activation method used, likely due to differences in composition and smaller particle size [67]. Furthermore, although Adseal and AH Plus share similar resin-based properties [95], a study indicates that their curing times are affected differently after direct UA for 20 seconds, potentially due to differences in their catalyst content [81]. Second, the selection of ultrasonic tip size. A study by Dash et al. (2017) utilized ultrasonic tips that were only one size smaller than the apical preparation size and observed a notable reduction in sealer penetration at certain root canal levels, potentially due to the increased contact between the ultrasonic tip and the canal walls in narrower regions. Conversely, other studies [38, 67] employing ultrasonic tips that were at least three sizes smaller than the apical preparation size have reported either an enhancement in sealer penetration or no statistically significant differences, without recording a marked decrease in penetration efficacy. Beyond tip size, there are variations in the positioning of the ultrasonic tip and the duration of activation. Despite these factors, consensus and standardization regarding the details of UA remain uncertain, underscoring the need for continued investigation into the specifics of ultrasonic application. Third, another aspect to consider is the utilization of the Lentulo spiral. Several studies [32, 35, 37, 38, 70] utilized a Lentulo spiral to place sealer into root canal. Research suggests that the rotational movement of the spiral can centrifugally disperse the sealer, propelling it toward the walls of the root canal and facilitating its penetration into the dentinal tubules [20, 59]. This introduces a confounding variable that warrants attention. Finally, regarding the use of the fluorescent dye Rhodamine-B, some research has raised doubts that the high moisture affinity of Rhodamine-B may lead to dye detachment from bioceramic sealers in the root canal's humid environment [96–98]. Additionally, the integration of Rhodamine-B has been shown to modulate the physicochemical attributes of endodontic sealers AH Plus and MTA Fillapex [99], potentially masking the true results.

Observation methods for penetration include confocal laser scanning microscope (CLSM), scanning electron microscopy (SEM), and stereomicroscope [88, 100]. CLSM employs fluorescent markers to indirectly visualize the sealing agent, enabling a perspective observation of its distribution within dentinal tubules [101]. In comparison to other techniques such as SEM and stereomicroscopy, CLSM eliminates the need for complex surface processing and mitigates issues arising from artifacts, debris, or sample loss [101, 101]. Despite its advantages, the accuracy of CLSM, particularly when utilizing Rhodamine-B as a fluorescent marker, has been questioned [98, 102, 103], as previously discussed. Consequently, it is recommended to further validate the distribution of Rhodamine-B-labeled bioceramic sealers and integrate and compare the findings from Confocal Laser Scanning Microscopy (CLSM) with other direct visualization techniques such as SEM [102, 104], potentially compromising penetration assessment accuracy.

Overall, no significant differences were observed in the average depth and area of sealer penetration, possibly due to various confounding factors. However, a meta-analysis on the percentage of sealer penetration revealed that ultrasound activation exhibited a favorable effect on sealer penetration (Fig. 4). Given the potential confounding factors and risk of bias in the current study, it is recommended that subsequent studies should comprehensively consider these elements to gain a deeper understanding of the potential benefits of UA.

### Push-out bond strength

Push-out test serves to evaluate the bonding between root canal sealers and the canal walls, reflecting the interaction between the sealer and dentinal walls [88]. There is conflicting evidence regarding the efficacy of UA in enhancing bond strength in this aspect of research.

The differences in research findings might be associated with several confounding factors, including the absence of sample size calculation [41, 50] and explanation of single operator [41, 50], insufficient standardization and storage of extracted teeth [41, 50, 105–107], challenges in controlling root canal moisture, and failure to match the plugger diameter to the specimen diameter ratio [41, 108, 109]. In particular, inconsistencies in the UA outcomes of EndoSequence BC sealer were evident in the studies by Chadgal et al.[50] and Carneiro et al. [70]. These discrepancies may be attributed to differences in root canal drying protocols, such as thorough drying versus no paper point drying. Notably, direct UA has been shown to improve the push-out bond strength of AH Plus, Sealer Plus BC, and Bio-C Sealer. Carneiro et al. [70] observed that 40 s of UA resulted in an improvement in the bond strength of these sealers, although statistical significance was not achieved in all instances. Similar effects were reported in cold lateral condensation studies by Tanomaru-Filho et al. [110], Wiesse et al. [28], De Bem et al. [30], and Jordani et al. [110]. Further physical–chemical research by Lopes et al. [81], Silva et al. [84], and Ames & So's et al. [84] supports these findings, demonstrating that UA improves the flowability and other properties of these sealers, thereby facilitating deeper penetration and enhancing their adhesion to dentin.

In summary, while there are inconsistencies in the overall evidence, it suggests that direct UA positively impacts the bond strength of AH Plus, Sealer Plus BC, and Bio-C Sealer sealers. Future studies should pay more attention to the control of confounding factors to further verify the effect of UA on different sealers.

## Conclusions

Based on a comprehensive analysis of the available evidence, it can be inferred that UA of sealers exerts a favorable influence on the filling quality during the single-cone obturation process. Convincing evidence has demonstrated the effectiveness of ultrasonically activated sealers in reducing void formation and enhancing the percentage of sealer penetration. However, inconsistent results regarding the average depth, area of penetration, and push-out bond strength, likely influenced by confounding factors and the limited number of studies, underscore the need for further investigation.

## Appendix A

Table: Details of the ultrasonic activation protocol

**Table:**

Types of Ultrasonic application	Author, Year	Apical preparation size	Ultrasonic activation protocol			
			Placement of tips	Activation time	Tip/sized	device/power
Indirect	Artingsih et al. 2021 [72]	30/07	against the cotton plier that holds the GP cone during the GP‘s insertion.	2~3s	tip (StartX #3, Dentsply-Maillefer)	device (P-5 Newtron XS; Satelec, Mount Laurel, NJ) set on “8” in the yellow code
	Kim et al. 2018 [23]	25/04 OR 30/04				
	Ko et al. 2020 [68]	30/09				
	Maharani, Ricardo, and Artiningsih 2021 [71]	40/06				
	Tanita et al. 2021 [41]	not mentioned				
	Moazami et al. 2020 [39]	30/09			tip (E7, NSK, Tochigi, Japan)	device (NSK, Tochigi, Japan) set on “8” in the yellow code
	Sfeir et al. 2023 [40]	25/08			tip (Start X #3) (Dentsply, Sirona, Bensheim, Germany)	not mentioned
	Kim et al. 2021 [69]	35/06			tip (StartX #3, Dentsply Maillefer)	device (Obtura-Spartan, Fenton, MO) set on the lowest power
	Maharani, Ricardo, and Artiningsih 2021 [71]	40/06			not mentioned*	device (P-5 Newtron XS; Satelec, Mount Laurel, NJ) set on “8”
	Kalantar Motamedi et al. 2021 [44]	30/varying degrees of taper			not mentioned	device (Woodpecker/DTE D5, Woodpecker Medical Instrument, Guilin, Guangxi, China) set on “G” mode ( the moderate power of the device).
	Gharechahi et al. 2024 [73]	30/04			tip (E7, Eighteeth, Jiangsu, China)	not mentioned
	Loiacono et al. 2024 [74]	25/08		3s×3^a^	not mentioned	not mentioned
Direct	Keles et al. 2023 [38]	40/06	within the canal, WL–2mm	20s	20/02 noncutting ultrasonic tip (EndoUltra, Micro Mega, Besançon, France)	an ultrasonic unit (EndoUltra)
	Alcalde et al. 2017 [32]	35/04		20s	Size 20 tip (Helse, São Paulo, SP, Brazil)	device (Jet Sonic; Gnatus, SP, Brazil) used at 20% of the power scale.
	Nikhil et al. 2015 [67]	50/05		20s	size 15 Satelec ultrasonic endodontic tip K15 Sonofile (Dentsply Tulsa)	The ultrasonic unit (Woodpecker DTe-D5 Ultrasonic scaler, China) used in endo mode
	Carneiro et al. 2023 [70]	50/05		40s	20/01 Tip (E1; Helse Ultrasonics)	Device (Piezon Master 200; EMS, Nyon) set to 03 (approximately 30% power)
	Drukteinis et al. 2021 [43]	50/06		10s	tip (Ultradent Products Inc., South Jordan, UT, USA)	Ultrawave XS (Ultradent Products Inc., South Jordan, UT, USA) at the medium power using Reflex technology, capable of automatic real-time frequency adjustment of 28–36 kHz.
	Chadgal et al. 2018 [50]	30/09		60s	size 15 ultrasonic K-file size 15 (SatelecActeon, USA)	piezoelectric ultrasonic handpiece (Satelec Acteon, USA) set at medium power
	Dash et al. 2017 [37]	30/09	within the canal, at WL	5s	size 25 ultrasonic endodontic tip K-25 Sonofile (Sonofile Dentsply Tulsa, USA)	Ultrasonic unit used in endo mode
	Coronas et al. 2020 [35]	35/06	within the canal	30s	Irrisonic ultrasonic tips (Irrisonic, Helse Technology, Ribeirão Preto, SP, Brazil)	not mentioned
	Damade et al.2020 [36]	30/09	not mentioned	not mentioned	not mentioned	not mentioned

Direct, the direct ultrasonic activation methods place the ultrasonic tips directly into the canal, primarily 2mm short of the working length. Indirect, the indirect ultrasonic activation methods place the ultrasonic tips against the cotton pliers holding the gutta-percha cone during insertion of the gutta-percha cone

*UA* ultrasonic activation, *NA* non-ultrasonic activation, *WL* working length, *GP *cone gutta-percha cone

^a^3s×3, Ultrasonic vibration was used for 3 periods, each period for 3 seconds

## Appendix B

Table: Characteristics of the sealers evaluated in the included studies

**Table:**

	Sealer	Manufacturer	Composition	Studies in which it was assessed
Calcium silicate-based sealers	Endoseal MTA	Maruchi, Wonju, Korea	Calcium silicates, calcium aluminates, calcium aluminoferrite, calcium sulfates, radiopacifier, and thickening agents	7 Artingsih et al. 2021 [72] Tanita et al. 2021 [41] Maharani, Ricardo, and Artiningsih 2021 [71] Damade et al.2020 [36] Moazami et al. 2020 [39] Kim et al. 2018 [23] Gharechahi et al. 2024 [73]
	EndoSequence BC/ iRoot SP/ TotalFill BC Sealer	Brasseler USA, Savannah, GA, USA/ Innovative BioCeramix, Vancouver, Canada/ FKG Dentaire, Chaux de Fonds, Switzerland	Zirconium oxide, calcium silicates, calcium phosphate monobasic, calcium hydroxide, filler, and thickening agents	5 Carneiro et al. 2023 [70] Sfeir et al. 2023 [40] Kim et al. 2021 [69] Artingsih et al. 2021 [72] Tanita et al. 2021 [41] Maharani, Ricardo, and Artiningsih 2021 [71] Chadgal et al. 2018 [50]
	Sealer Plus BC	MK Life, Porto Alegre, RS, Brazil	Calcium disilicate, calcium trisilicate, zirconium oxide, calcium hydroxide, propylene glycol	2 Carneiro et al. 2023 [70] Coronas et al. 2020
	Endoseal TCS	Maruchi, Wonju, Korea	Tricalcium silicate, zirconium dioxide, dimethyl sulphoxide, thickening agent	2 Ko et al. 2020 [68] Gharechahi et al. 2024 [73]
	Bio-C Sealer	Angelus, Londrina, Paraná, Brazil	Calcium silicates, calcium aluminate, calcium oxide, zirconium oxide, iron oxide, silicon dioxide, dispersing agent	2 Carneiro et al. 2023 [70] Loiacono et al. 2024 [74]
	BioRoot RCS	Septodont, Saint Maur-des-Fosses, France	Powder: tricalcium silicate, zirconium dioxide, and povidone. Liquid: water, calcium chloride, and polycarboxylate	1 Drukteinis et al. 2021 [43]
	MTA Fillapex	Angelus, Londrina, Brazil	Paste A: salicylate resin, bismuth trioxide, fumed silica, Paste B: fumed silica, titanium dioxide, mineral trioxide aggregate, and base resin.	1 Nikhil et al. 2015 [67]
	Sure-Seal Root sealer	Sure-endo, Sagimakgol-ro, Jungwon-gu, Seongnam-si, Korea	Zirconium, bioactive glass, glass ceramic, radiotherapy glass, composites of hydroxyapatite, resorbable calcium phosphate	1 Keles et al. 2023 [38]
	GuttaFlow Bioseal	Coltene Holding AG, Altstätten, Switzerland	Gutta-percha, polydimethylsiloxane, zinc oxide, barium sulfate, zirconium oxide, platinum catalyst, color pigments, micro silver, bioactive glass ceramic	1 Damade et al.2020 [36]
Resin epoxy-based sealer	AH Plus	Dentsply DeTrey GmbH, Konstanz, Germany/Dentsply Maillefer, Ballaigues, Switzerland	Paste A: bisphenol A/F epoxy resin, calcium tungstate, zirconium oxide, silica, iron oxide Paste B: adamantane amine, N,N-dibenzyl-5-oxanonane, TCD-diamine, calcium tungstate, zirconium oxide, silica, silicone oil	3 Carneiro et al. 2023 [70] Alcalde et al. 2017 [32] Nikhil et al. 2015 [67]
	Adseal	Metabiomed, Cheongju, Korea	Poly(1,4-butanediol) bis(4-aminobenzoate) (30–40%), Bisphenol A diglycidyl ether–bisphenol A copolymer (20–30%), 2-Hydroxyethyl salicylate (15–20%), Triethanolamine (<10), calcium oxide (<5%).	2 Kalantar Motamedi et al. 2021 [44] Dash et al. 2017 [32]
